# Flow-Seq Method: Features and Application in Bacterial Translation Studies

**DOI:** 10.32607/actanaturae.11820

**Published:** 2022

**Authors:** E. S. Komarova, O. A. Dontsova, D. V. Pyshnyi, M. R. Kabilov, P. V. Sergiev

**Affiliations:** Institute of Functional Genomics, Lomonosov Moscow State University, Moscow, 119234 Russia; Department of Chemistry, Lomonosov Moscow State University, Moscow, 119234 Russia; Skolkovo Institute of Science and Technology, Moscow, 121205 Russia; Belozersky Institute of Physico-Chemical Biology, Lomonosov Moscow State University, Moscow, 119234 Russia; Shemyakin-Ovchinnikov Institute of Bioorganic Chemistry, Russian Academy of Sciences, Moscow 117437 Russia; Institute of Chemical Biology and Fundamental Medicine, Siberian Branch of the Russian Academy of Sciences, Novosibirsk, 630090 Russia

**Keywords:** Flow-seq, NGS, high-throughput sequencing, flow cytometry, translation, bacteria

## Abstract

The Flow-seq method is based on using reporter construct libraries, where a certain element regulating the gene expression of fluorescent reporter proteins is represented in many thousands of variants. Reporter construct libraries are introduced into cells, sorted according to their fluorescence level, and then subjected to next-generation sequencing. Therefore, it turns out to be possible to identify patterns that determine the expression efficiency, based on tens and hundreds of thousands of reporter constructs in one experiment. This method has become common in evaluating the efficiency of protein synthesis simultaneously by multiple mRNA variants. However, its potential is not confined to this area. In the presented review, a comparative analysis of the Flow-seq method and other alternative approaches used for translation efficiency evaluation of mRNA was carried out; the features of its application and the results obtained by Flow-seq were also considered.

## INTRODUCTION

Translation is the key process in the vital activity of all organisms, during which proteins are synthesized in cells using a macromolecular ribonucleoprotein complex known as the ribosome. It decodes the information in mRNA and translates it into the sequence of amino acids that form the protein [1]. Moreover, not only does mRNA participate in this process as a passive information carrier, but it also predetermines the translation efficiency [2].

**Fig. 1:**
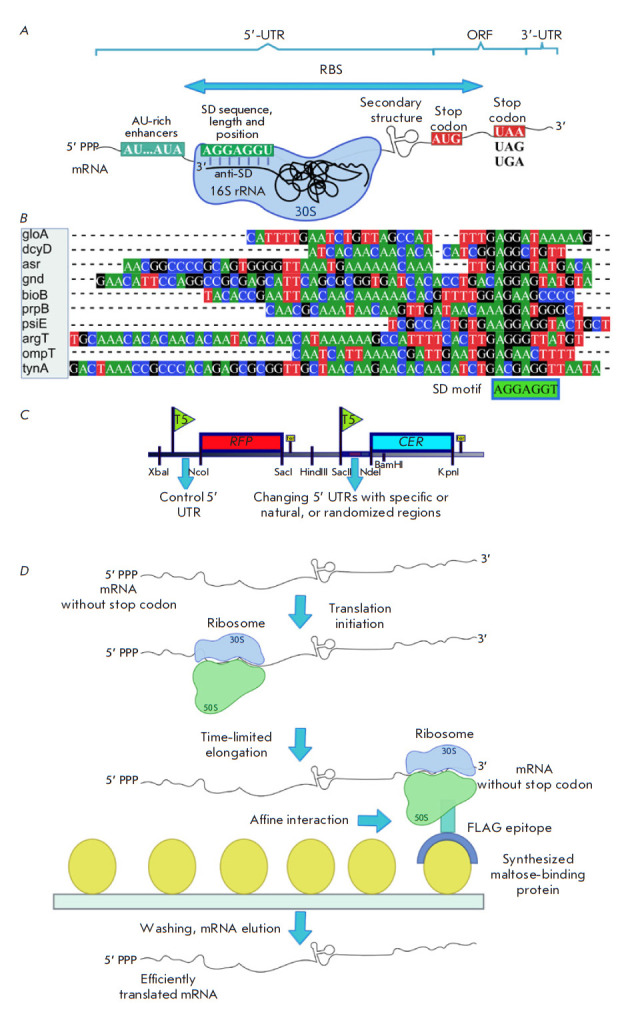
(*A*) – Structural features of mRNA in bacteria. 5’ and 3’ UTR – the 5’ and 3’ untranslated regions, respectively. RBS – the ribosome binding site on mRNA. ORF – the open reading frame containing the protein-coding sequence. SD and anti-SD – Shine–Dalgarno and anti-Shine–Dalgarno sequences, respectively. (*B*) – An example of 5’ UTR mRNA sequence alignment used in a large-scale analysis of untranslated gene regions with the SD motif highlighted. (*C*) – An example of a dual-reporter construct with control 5’ UTR upstream of the RFP fluorescent protein gene and a variable 5’ UTR upstream of the second CER fluorescent sensor protein gene to assess the effect of the features of the variable region on the translation efficiency. (*D*) – The scheme of affinity isolation of ribosomes with efficiently translated mRNA. Selection was carried out by limiting the time of *in vitro *translation. The mRNA contains 5’ UTR, the coding region that includes the region encoding the FLAG epitope interacting with the synthesized maltose-binding protein and TolA allowing the epitope to exit the ribosome tunnel and fold properly. There is no stop codon in the mRNA construct, so the ribosome remains on it. The drawing was executed in the Inkscape software

The 5’ untranslated region (5’ UTR) of mRNA is one of the sites responsible for its translation efficiency (Fig. 1A) [3]. The 5’ UTR contains the ribosome binding site (RBS) carrying the Shine–Dalgarno (SD) sequence [4, 5, 6, 7, 8, 9, 10, 11, 12, 13] complementary to the 3’ terminus of 16S rRNA in canonical mRNAs [14, 15]. To ensure high efficiency of the protein synthesis, the SD sequence needs to be located at an optimal distance from the start codon and have an optimal length [16, 17, 18]. Sometimes a single 5’ UTR can carry several Shine–Dalgarno sequences [2, 17]. For efficient translation, the translation initiation region (TIR) needs to be either fully single-stranded or folded into the secondary structure that can be easily disturbed [19, 20, 21, 22]. Other elements capable of affecting the translation efficiency are known, such as the adenine- and uracil-rich (AU-rich) mRNA region that the ribosomal protein bS1 interacts with [23, 24, 25], as well as the initial portion of the coding region immediately downstream of the start codon [26, 27, 28]. The 5’ UTRs of efficiently translated mRNAs are characterized by low abundance of cytidine residues and the presence of purine repeats (AG repeats) [2].

Today, there are various methods that allow one to study the functional significance of individual mRNA sites for protein synthesis. These methods involve site-directed mutagenesis [29] or randomization [30, 31] of 5’ UTR motifs (usually upstream of the fluorescent protein gene), and assessment of its fluorescence intensity in vitro (or in vivo), which is indicative of translation efficiency. The in silico thermodynamic simulations [18, 32, 33, 34, 35, 36], which estimate the strength of molecular interactions between the 30S complex and the mRNA transcript and predict the translation initiation rate, can be used to determine the values related to the translation efficiency. The simulation results can be selectively verified experimentally using reporter constructs. The emergence of the flow cytometry method has made it possible to simultaneously assess different parameters of a large number of cells in vivo and isolate individual fractions based on the similarity of certain parameters (e.g., according to the expression level of the fluorescent protein gene) [37]. Advancements in next-generation sequencing (NGS) have contributed to the development of novel, comprehensive approaches to genome research and to the determination of the genotype–phenotype correlation (e.g., whole genome sequencing, sequencing of plasmid DNA libraries, RNA sequencing for single-cell transcriptome profiling and isolation of efficiently translated mRNA, as well as ChIP sequencing for identifying the binding sites of DNA-associated proteins) [38, 39].

## THE VARIETY OF APPROACHES TO STUDYING THE ROLE OF 5’ UTRS IN TRANSLATION EFFICIENCY

Comprehensive analysis of E. coli genes has shown that most mRNAs carry the Shine–Dalgarno (SD) sequence (Fig. 1B), which was discovered in several bacterial mRNAs in the 1970s [4] and is essential for efficient translation initiation [16, 17, 18]. The SD sequence is the best studied regulatory element. It resides 5–8 nucleotides upstream of the start codon (or 8–11 nucleotides when starting counting from the central G base in the SD sequence [7]) and acts as a binding site to the bacterial 30S subunit, unlike in the eukaryotic ribosome, which binds to the 5’ terminus of mRNA for scanning initiation [6]. Different E. coli mRNAs contain SD sequences of different lengths, varying between four and eight nucleotides. The most plausible composition of the SD sequence is agGa.

The dependence between the protein synthesis efficiency and the length of the SD sequence and its distance from the start codon was studied using various methods (e.g., using a dual genetically engineered construct Fig. 1C) carrying the genes of two fluorescent proteins, where one of the proteins, RFP (red fluorescent protein), was an internal control and the other one, CER (cyan fluorescent protein), acted as a sensor of the effects associated with variations in the mRNA 5’ UTRs) [17]. The ratio between the measured fluorescence intensities of the two proteins (CER/RFP) in vivo was calculated, making it possible to neutralize the effects caused by the bacterial cell size and fluctuations in the abundance of the reporter plasmid. This approach, based on molecular cloning with the use of 16 reporter constructs with four SD sequences (2, 4, 6 and 8) of different lengths residing at different distances from the start codon of the CER protein gene (7, 10, 13 and 16) and another control construct carrying no sites complementary to the anti-SD sequence, allowed the researchers to experimentally study the effect of the SD sequence length, the distance between the SD sequence and the start codon, and their combinations on the synthesis of the CER protein. Therefore, it was demonstrated that the translation efficiency of mRNA carrying the 8-nucleotide SD sequence declines with increasing distance between the start codon and the SD sequence. For the 6-nucleotide SD sequence, the optimal distance is 10 nucleotides. The same dependence was observed for the medium-length SD sequence (four nucleotides), as in the case of a long SD sequence (eight nucleotides). For the short SD sequence (two nucleotides), the effect of distance was negligible, while the role of this SD sequence in the protein synthesis efficiency was preserved: it ensured an efficiency that was one order of magnitude greater than that when using the control construct without the SD sequence. By varying these parameters, one can change the translation level by up to four orders of magnitude, which indicates that they are important for determining the level of many proteins in the cell [17].

Numerous variants of the motif in 5’ UTR produced by site-directed mutagenesis based on use of the polymerase chain reaction (PCR) can be employed to perform a rapid, and fairly simple, quantitative analysis of gene expression in vitro. The PCR product containing the T7 promoter sequence, the tested 5’ UTR variant, and the eGFP fluorescent protein gene are directly used in the coupled transcription– translation in vitro system from E. coli cells [29]. The translation efficiency in this system can be assessed according to eGFP fluorescence intensity. This method was used to produce 54 variants of 5’ UTR sequences (18 and 36 of those having modified SD- and AU-rich sequences, respectively), which ensured a 0.1–2.0 range of relative expression levels and revealed the effects of different ribosome binding sites (RBSs) on the translation efficiency [29]. However, this pointwise approach is substantially confined to the small set of variants being tested, making it impossible to apply it to the entire variety of natural 5’ UTRs lying upstream of the genes (their number in E. coli being ~ 4 × 10^3^) [8].

An experimental system (Fig. 1D) [30] based on in vitro translation was subsequently developed, which allowed one to select the most efficiently translated mRNAs from a large sample of synthetic sequences. A model mRNA containing an 81-nucleotide 5’ UTR was used for this purpose; 18 of these nucleotides, residing upstream of the start codon, were completely randomized: so, a library consisting of ~6.9 × 10^10^ different sequences was successfully obtained. The model mRNA encoded a fusion protein containing a maltose-binding domain approximately in its center and the FLAG epitope, which allowed one to perform affinity purification of the ribosomes that synthesized this fusion protein. The TolA protein fragment resided downstream of the domain used for affinity purification; this fragment acted exclusively as a spacer sufficient for affinity domain exposure from the peptide tunnel once the full-length fusion protein was synthesized. This mRNA did not carry the stop codon; therefore, it remained bound to the ribosome after the synthesis had been completed in that experiment. Therefore, mRNA could have been extracted from affinely bound ribosomes and subsequently amplified. The limited translation time was the key parameter of mRNA selection: only rapidly translated mRNAs could be affinely purified and used in the next selection round [30]. Surprisingly, 76% of the selected sequences ensuring the most rapid translation in the in vitro system carried no SD sequences and had C-rich short sites complementary to 16S rRNA. However, a high expression level of mRNAs with these C-rich sequences was not observed in vivo, which, potentially, was caused by different average ratios in the in vitro and in vivo ribosomal systems and mRNAs, which competed with C-rich RBS for ribosome binding [30]. The same experiment was conducted using a library of shorter mRNAs with a 40-nucleotide 5’ UTR [31], which are the most abundant in E. coli mRNA [40, 41]. Next-generation sequencing and statistical tools made it possible to identify the mRNA–ribosome binding motifs. The mRNAs selected from a library with shorter 5’ UTRs according to the translation rate were more likely to contain SD sequences, along with G/U-rich ones [31]. The results of this study are also indicative of the fact that the 5’ UTR length affects the efficiency of protein synthesis initiation.

The sequence of mRNA 5’ UTRs can be responsible for folding variations in the region upstream of the start codon. The association between the stability of the secondary structures in the TIR and the translation efficiency was confirmed by a large-scale computational analysis [19], which revealed that prokaryotic and eukaryotic genes, especially those characterized by high expression levels, tend to destabilize the mRNA secondary structure near the start codon [20]. By varying the stability ( < -12 kcal/mol) of the hairpin structure carrying the RBS by site-directed mutagenesis, followed by an in vivo analysis of the protein yield, it was discovered that the higher the stability of the secondary structure carrying the ribosome binding site, the lower the translation efficiency. Therefore, it has been demonstrated that it is possible to vary the expression 500-fold by making a single nucleotide substitution, which stabilizes the mRNA secondary structure. As a result, translation initiation was entirely dependent on the spontaneous unfolding of the entire mRNA initiation site [21]. However, this spontaneity had to do with the fact that all the essential elements of the initiation complex were present [22]. This analysis of 12 mRNAs characterized by different levels of secondary structure stability and carrying SD sequences of different lengths (or without SD sequences) revealed that the SD sequence per se, the start codon, the initiator tRNA with formylated methionine, and the GTP-bound translation initiation factor 2 (IF2), in a complex with the 30S ribosomal subunit, are required for the unfolding of the mRNA secondary structure. The contribution of each individual element to the disruption of TIR mRNA folding process was assessed using the dissociation constant of the mRNA fragment carrying a 6-nucleotide SD sequence [22]. FRET analysis of the same fragment labeled with Cy3 and Cy5 at the 5’ and 3’ termini, in the presence of the 30S subunit and all other elements required for translation initiation, was subsequently conducted. The assessment was performed with respect to control mRNA that carried no SD sequence but whose secondary structure was characterized by a similar level of stability. The analysis revealed the significant role played by the SD sequence in the unfolding of the mRNA secondary structure. FRET analysis was shown to be highly efficient for the folded mRNA whose termini were involved in a complementary interaction between the SD and anti-SD sequences; poor efficiency of the FRET analysis was demonstrated for the unfolded mRNA [22].

**Fig. 2:**
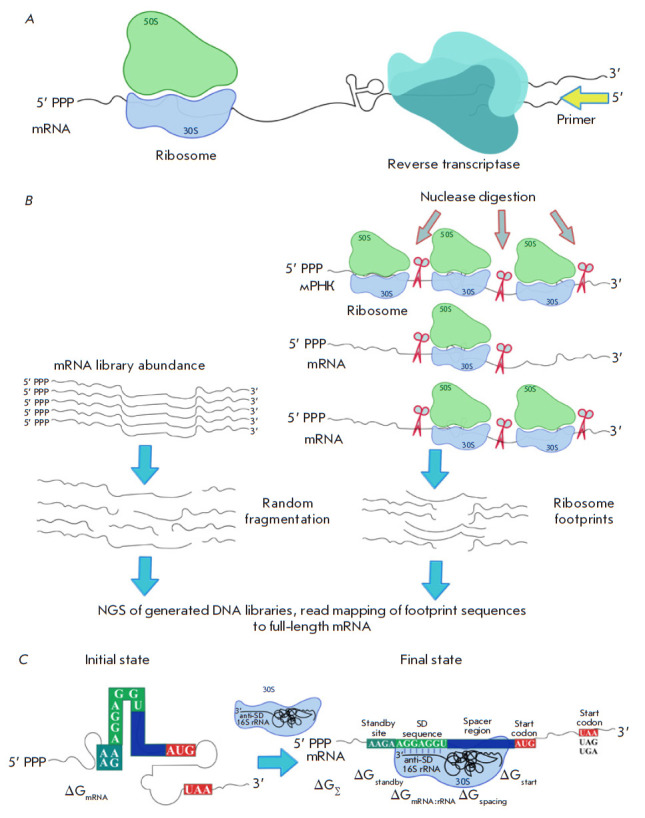
(*A*) – The principle of the toeprinting technique. Stable ribosome complexes stop reverse transcriptase at a specific mRNA position, thus generating short cDNA products of a specific length. Primers for reverse transcriptase can be radioactively or fluorescently labeled. (*B*) – The scheme of the ribosome profiling method (Ribo-seq). After translation initiation, mRNA is cut by a specific nuclease at the sites where it is not protected by ribosomes. In parallel, the original mRNA library is prepared for sequencing by random fragmentation. It will be used as a reference sequence. All obtained ribosome footprints are used to prepare a DNA library, which is further deeply sequenced. Based on the NGS results, footprint sequence reads are mapped to full-length mRNA. (*C*) – The thermodynamic model of bacterial translation initiation. Changes in free energy during the initiation stage depend on the five types of molecular interactions defining the initial and the final states of the system. The drawing was executed in the Inkscape software

The efficiency of the binding of ribosomal subunits to a particular 5’ UTR mRNA sequence is assessed using the so-called toeprinting method (Fig. 2A). It is based on the use of fluorescent- or isotope-labeled primers complementary to the 3’ terminus of mRNA. The reverse transcription reaction is performed after the assembly of the initiation complex on mRNA, followed by an electrophoretic analysis of elongated cDNA in the reaction mixture. Reverse transcriptase reaches the 5’ terminus of mRNA if it is not bound to the ribosome and forms shorter products in the case when reverse transcriptase stops once it has encountered the ribosome. The ratio between long and truncated toeprints allows one to estimate the proportion of mRNAs bound to the ribosome [42, 43].

As the experimental results are acquired and methods for analyzing them are developed, bioinformatic approaches allowing one to work with large datasets start to play an increasingly important role. Translation initiation of prokaryotic mRNAs (where the SD sequence has not been detected in 5’ UTR) observed in the experiments occurs independently of any interactions with the anti-SD sequence and is mediated by the ribosomal protein bS1. A bioinformatic analysis showed that the stability of the secondary structures of such 5’ UTR sequences was reduced, thus facilitating the formation of the initiation complex and compensating for the lack of SD and anti-SD interactions [44, 45].

There exist the so-called prokaryotic leaderless mRNAs, which carry neither the 5’ UTR nor the SD sequence. However, a large-scale in silico analysis of the macroevolution revealed that the number of such genes in bacteria has declined over time. The translation initiation sites of all the genes in 953 bacterial and 72 archaeal genomes have been examined and categorized into groups, according to the distance to the root (between bacteria and archaea) on the 16S rRNA-based phylogenetic tree. The average proportion of leaderless genes in each group was calculated: first, it drops rapidly and subsequently fluctuates at a low level [46].

Intense development of next-generation sequencing methods and the accumulated skills in working with the translation system have made it possible to develop the ribosome profiling (Ribo-seq) method (Fig. 2B), which is based on high-throughput sequencing of mRNA fragments protected by the translating ribosome [47]. This approach proved to be effective for studying gene expression, simultaneously at both the transcriptional and translational levels, including in response to various impacts [48 , 49, 50]. The Ribo-seq technique provides information about the location of ribosomes on mRNA with a single-nucleotide resolution. This accuracy allows one to detect translation of mRNA sites outside of the annotated reading frame, as well as detect translation of the overlapping reading frames and semantic stop codon decoding. The translatable reading frames were identified using Ribo-seq in RNAs that had been previously considered non-coding. It was also possible to evaluate the effect of various conditions and factors on mRNA translation in cells (e.g., different environments, modifications of proteins and antibiotics) [51 , 52, 53, 54, 55, 56].

The extensive use of the Ribo-seq method has unearthed a number of challenges and artifacts related to the experimental methodology and data analysis [57, 58, 59]. The promising ribosome profiling technique used to study the ribosome decoding rate is characterized by infrequent high peaks in the ribosome footprint density and by long alignment gaps of the respective mRNA sequences. In order to reduce the impact of data heterogeneity, a normalization method has been elaborated. This method is efficient in the presence of heterogeneous noise and has revealed significant differences in read distribution across mRNA and the determinants of ribosome footprint frequencies in 30 publicly available ribosome profiling datasets, thereby casting doubt on the reliability of this method as an accurate representation of local ribosome density without prior quality control [57]. This observation suggests an incomplete understanding of how the protocol parameters affect the ribosome footprint density.

The most likely reason for this observation probably consists in the sequence shifting that occurs during the construction of the ribosome footprint library and its conversion into cDNA, followed by sequencing [58]. The aforementioned steps involve a number of reactions using sequence-specific enzymes such as nucleases [60]. Meanwhile, some antibiotics used to treat ribosomes prior to profiling have the same sequence specificity [61, 62, 63], which must be taken into account in experiment setting.

It has been shown using ribosome profiling in bacteria that ribosome occupancy downstream of the Shine–Dalgarno sequences occurring randomly in the coding region is significantly increased [64]. Whereas the SD sequences upstream of the start codon play a well-characterized role in translation initiation, the findings indicate that elongation is slowed down by the formation of transient base pairs between the SD motifs within the open reading frames and the anti-SD sequence in 16S rRNA, such pauses accounting for over 70% of the strong pauses throughout the genome; they are considered to be the main determinant of translational pausing in bacteria [64].

Later, the modified high-resolution Ribo-seq method was used to demonstrate that the previously observed enrichment of the ribosome occupancy at  the SD motifs can be attributed to pauses at glycine codons and the impossibility of isolating the entire population of ribosome-protected mRNA fragments. A conclusion has been drawn that the SD motifs are probably not the main cause of the multiple pauses noted during translation in vivo [65].

The biophysical models allow one to assess the efficiency of biomolecule interactions, including the mRNA–ribosome ones. The thermodynamic model can be used as an example (Fig. 2C) [32]; this model simultaneously estimates the strength of the molecular interactions of the 30S complex with the mRNA transcript, calculates the Gibbs free energy for each element within a particular mRNA, and predicts the translation initiation rate: the higher energy needs to be spent to unfold mRNA elements, the lower the translation initiation rate is. The presented model can be used both to predict the relative translation initiation rate of an existing 5’ UTR with the identified RBS and to design an RBS sequence ensuring the required translation initiation rate [18, 32].

The Flow-seq method used for a library of plasmids carrying the fluorescent protein genes (the first one acting as an internal control, and expression of the second varying depending on the impact of the sequences obtained by randomization of 30 nucleotides in the coding region of the gene immediately downstream of the start codon) allowed one to divide the resulting library (over 30 × 10^3^ mRNA variants) according to translation efficiency [28]. Further analysis showed that the translation efficiency of mRNAs carrying the SD-like sequences was reduced, and that the proportion of such mRNAs in the set of efficiently translated mRNAs also declined, being indicative of the negative effect of the SD sequences in this mRNA region on the protein synthesis and, in turn, supporting earlier findings obtained for a limited set of model mRNAs [66].

Interestingly, the distribution of the binding energies of the anti-SD sequences among efficiently translated mRNAs is similar to that in natural E. coli genes. Moreover, individual constructs carrying the SD sequences in the sliding window of the initial coding region immediately downstream of the start codon and having similar energies of secondary structure folding have been designed, and their translation efficiency has been evaluated. Hence, the findings obtained are consistent with the results of the data analysis performed after using the Flow-seq method [28].

## THE SCHEME OF THE FLOW-seq TECHNIQUE, THE FEATURES AND RESULTS OF ITS APPLICATION

**Fig. 3:**
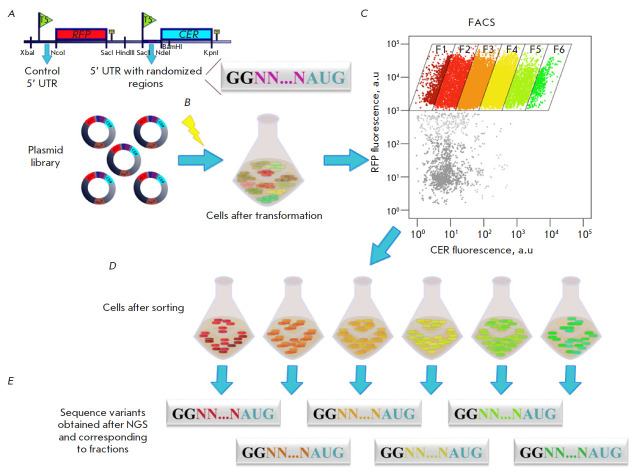
The scheme of the Flow-seq method (as exemplified by working with randomized 5’ UTR upstream of the CER protein gene and control 5’ UTR upstream of the RFP protein gene). The stages of plasmid library construction, transformation, sorting, and sequencing are presented. (*A*) – Cloning of a randomized DNA fragment into a reporter vector upstream of the CER protein gene. A constant 5’ UTR is retained upstream of the RFP protein gene. (*B*) – Electroporation of the entire plasmid library into *E. coli *cells. (*C*) – Cell separation based on the CER/RFP fluorescence intensity ratio by a cell sorter. (*D*) – Cell fraction collection (e.g., F1–F6) according to the CER/RFP ratio. (*E*) – DNA isolation and randomized region amplification followed by high-throughput sequencing (NGS). The drawing was executed in the Inkscape software

Thousands of reporter constructs are often used to determine the effect of a certain factor or a set of factors on the expression level of a particular gene by sorting various promoter variants, 5’ untranslated regions, and the individual sites in them (including the ribosome-binding sites (RBSs), the upstream regions (standby sites) or the downstream spacer sites), as well as the initial ramp regions of the coding sequence, either individually or simultaneously (Table 1). These plasmids typically carry two fluorescent protein genes: the first one acting as a sensor whose expression is sensitive to variable sites and the second one being used as an invariant internal control. The resulting sets of constructs are used to transform the bacterial strain suitable for further expression and sorting. Next, the fluorescence intensities of the two proteins in the cell pool are estimated using flow cytometry and cell groups/fractions characterized by approximately identical ratios of the measured fluorescence levels of these proteins are formed. Once the number of collected cells is increased, plasmids are isolated from the cells; the variable site is amplified and subjected to high-throughput sequencing in order to determine the DNA/RNA sequences in the particular fraction ensuring a particular level of reporter gene expression (Fig. 3).

This approach was applied to design a number of constructs simultaneously carrying different combinations of ribosome binding sites and promoters. The amounts of RNA and green fluorescent protein (GFP) synthesized by the cells transformed with each construct were compared to the amount of respective DNA, thus determining the transcription and translation efficiencies. The mCherry fluorescent protein gene, which was used as an internal control and carried a conserved promoter and ribosome binding site (RBS), was also inserted into the construct [67]. A set consisting of 12,653 plasmids with various combinations of 114 promoters and 111 RBS variants was eventually obtained. In order to estimate the steady-state DNA and RNA levels, deep sequencing of DNA (DNA-seq) and RNA (RNA-seq) from the cells in this phase was carried out. To assess the levels of the two fluorescent proteins, the cells were sorted according to the ratio between the GFP/mCherry fluorescence intensities. Plasmid DNA was isolated from cell populations with similar GFP/mCherry fluorescence intensity ratios and subjected to deep sequencing. The extracted sequences belonging to a particular group were tagged with group-specific barcode sequences, which were further used for searching for and sorting sequences into previously defined groups during the analysis of sequencing reads. The levels of two fluorescent proteins in the groups were then assessed; the GFP/mCherry ratio was defined as a measure of translation efficiency; the cells were subdivided into three types according to this ratio: weak, medium and strong, and the corresponding sequences were identified. As anticipated, the cells in the library contained approximately identical levels of the mCherry protein, whose fluorescence intensities were characterized by the normal (Gaussian) distribution and varied within one order of magnitude, whereas the expression levels of the gfp gene varied by four orders of magnitude. A total of 282 individual colonies were verified by sequencing; 55% of these colonies were appropriate (i.e., contained error-free invariable sites, and the expected promoter variants and ribosome binding sites were identified for them without mutations). The fluorescence levels of most of these 55% appropriate promoter and RBS combinations were measured and subsequently used as a control set.

**Table 1:** Application of the Flow-seq method to the analysis of the translation efficiency

mRNA elements	Number of variants in the generated libraries after Flow-seq	Variant types	Results	Reference
Promoters and ribosome binding sites (RBS) in 5’ UTR	11,894 (94%) out of 12,653 possible variants with combinations of 114 promoters and 111 RBSs (one combination resulted in the incompatible restriction site)	Taken from the available databases and generated using the RBS Calculator	The range of expression variations - four orders of magnitude. Promoter choice has the greatest effect on the RNA level and a smaller one on protein level, since its translation efficiency is also affected by the choice of the ribosome binding site and, potentially, other factors. 55% out of several hundreds of tested individual colonies were unmistakably identified during the Flow-seq analysis	[67]
Promoters and ribosome binding sites (RBS) in 5’ UTR	~ 500 combinations of 14 promoters and 22 RBSs for two detectable fluorescent proteins and more than 1,200 combinations from the randomized library	Specific variants and variants with randomized sequences in the elements under study	The dynamic range of expression - three orders of magnitude. The resulting combinations lead to the expression of a random gene (twofold variation in the expression level) with 93% reliability	[75]
Six nucleotides in the spacer region downstream of the SD sequence in the 5’ UTR and upstream of the start codon and the first six nucleotides following it (codons at positions +2 and +3 of the coding sequence (CDS)) …-SD-GAC-6N-AUG-6Nsyn-…	13,914 (56%) variants for one protein and 25,861 (53%) variants for another protein out of 24,576 and 49,152 possible variants, respectively	Randomized spacer regions and codons at positions +2 and +3 with synonymous substitutions not changing the coding sequence of two sensor proteins	The range of expression variations - three orders of magnitude. The low GC-content and reduced stability of the secondary structure of the studied elements are important for the high expression level not limited by these determinants. The distribution of the protein fluorescence levels measured in several dozen colonies using a plate reader is consistent with the Flow-seq data	[71]
Four nucleotides in the spacer region downstream of the SD sequence in the 5’ UTR and upstream of the start codon …-SD-C-4N-CAU-AUG-…	249 (97%) out of the 256 possible variants	Randomized	The range of expression variations - two orders of magnitude. The predominant adenosine content and reduced cytidine content in efficiently translated variants. The low GC-content and reduced stability of the secondary structure of the studied elements are important for a high expression level. The SD-like sequences also occur only in the highly expressed variants	[39]
Six-nucleotide SD sequence in the 5’ UTR	4,066 (99%) out of the 4,096 possible variants	Randomized	The measured levels of proteins (fluorescent and five natural ones) for 91% of the sequence variants lay within the twofold range of variations in the expression level predicted using the EMOPEC tool that takes into account the context of the SD sequence, which minimized variations in the secondary structure	[76]
Standby sites of different lengths (20–164 nucleotides) upstream of the SD sequence, distal in the 5’ UTR	136 5’ UTRs with different lengths and secondary structures, shapes, and number of modules	Modeled variants	The range of variations in translation efficiency - two orders of magnitude. The rate of mRNA translation initiation is controlled by the surface area of single-stranded regions, partial unfolding of the RNA structure for minimizing the ribosome binding free energy penalty; there is no cooperative binding and, possibly, ribosome sliding in the analyzed region. The biophysical model for predicting the translation initiation rate has been developed and experimentally tested. The ribosome can easily bind to the modules of standby sites that are remote from the start codon and ensure high translation efficiency	[34]
The ribosome binding site (RBS) in the 5’ UTR with a fixed SD sequence (five nucleotides) and the variable standby site (four nucleotides) and the six-nucleotide spacer region RRRV-AGGAG-R-6NAUG (R: A/G, V: A/G/C, N:A/U/C/G)	More than 20,000 (10%) out of ~ 200,000 possible variants for two fluorescent proteins	Randomized and partially specific positions with incomplete variations	The range of variations in translation efficiency - four orders of magnitude. The translation efficiency is significantly affected by conservation of the SD sequence, whereas the AC-rich spacer region is weakly dependent on the context. Low stability of the secondary structure of the studied region was observed for high expression. Replacement of the reporter protein with another one often had no effect on the overall trend in the distribution of the sequences defining a given protein synthesis level	[74]
Almost complete 5’ UTR sequence (22 or 32 nucleotide long) GG-20N/30N-AUG…	11,692 (10-6% out of the possible variants), 11,889 (10-12%) for 20N and 30N, respectively; 48 natural variants with variations	Randomized, natural, and specific	The range of variations in translation efficiency - four orders of magnitude. Low stability of the secondary structure and conservation of the SD sequence in highly expressed variants were observed. The presence of AU-rich enhancers at the 5’ terminus in the standby site, the low cytidine content, multiple SD sequences, and AG repeats in mRNA 5’ UTRs ensure high translation efficiency in a number of cases	[2]
5’ UTR sequences (2–60 nucleotides long) of the first genes of E. coli operons with GG at the 5’ terminus retained during transcription GG-natural 5’ UTR	648 (91%) out of the 713 possible variants 2–60 nucleotide long, (45%) out of all the 1,451 natural 5’ UTRs of the first operon genes	Natural	The range of variations in translation efficiency - 30-fold. The RNA secondary structure and SD sequence affected the translation efficiency, but with lower variability compared to the randomized libraries. The low secondary structure stability and conservation of the SD sequence in highly expressed variants. The results of an estimation of the translation efficiency for individual 5’ UTRs correlated with the ribosome profiling data	[77]
Sites in the promoter region, the standby site 10/20/30 nucleotides long, the 8-nucleotide spacer region 10N/20N/30N-SD-8N	~ 12,000 (a very small percentage of the possible variants)	Randomized	The range of variations in translation efficiency - five orders of magnitude. At a high expression level, low stability of the secondary structure of the studied region was observed	[72]
Promoters, ribosome binding sites (RBS), the first 13 amino acids of the protein-coding region	14,234 combinations of two promoters, four ribosome binding sites (RBSs), and sequences of N-terminal peptides corresponding to the first 13 amino acids in 137 natural E. coli genes	Natural	The range of variations in translation efficiency - more than two orders of magnitude. The use of rare codons at the N-terminus can increase expression 14-fold regardless of RBSs, ensuring a degree of translation efficiency. Reduction of secondary structure stability, rather than codon rarity itself, is responsible for increasing the expression level	[78]
The first six codons downstream of the start codon in the coding sequence	10	Natural	Reduction of secondary structure stability, rather than codon rarity itself, is responsible for increasing translation efficiency. Rare codons are often A/T-rich at position 3, which is more likely to correlate with increased expression than the synonymous G/C-ending codons	[81]
The first 10 codons downstream of the start codon in the coding sequence	More than 30,000	Randomized	Reduction of secondary structure stability, rather than codon rarity itself, is responsible for increasing translation efficiency. Codons located closer to the start codon have a significant effect on expression. Additional start codons in the reading frame facilitate translation. The presence of amino acids for the synthesis of which the cell expends a lot of resources, in the N-terminal motif of the protein negatively affected protein synthesis efficiency	[28]

**Fig. 4:**
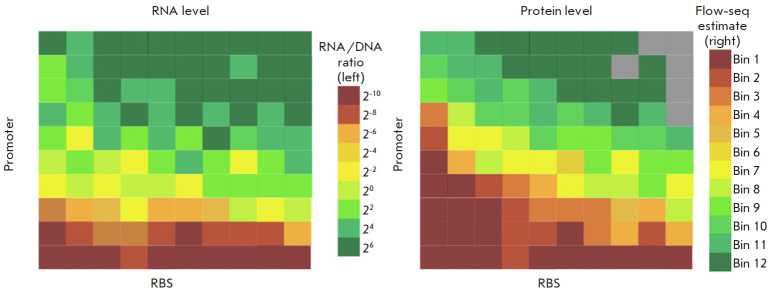
A schematic image of the exemplary representative maps of RNA and protein synthesis efficiency levels. RNA (left) and protein (right) levels for a small set of constructs are gridded according to the identity of the promoters (the Y axis) and ribosome binding sites (RBS, the X axis). Promoters and RBSs are sorted in ascending order of the average efficiency of RNA and protein synthesis, respectively. Gray cells indicate constructs corresponding to levels below an empirically defined threshold. Scales of RNA levels (the RNA to DNA ratios) and protein levels (ratios of GFP (green) to RFP (red) fluorescence proteins) are shown to the right of their respective maps. The drawing was executed based on the source [67] in the Inkscape software

The results obtained by large-scale sequencing of DNA and RNA and the measured gene expression levels of the fluorescent proteins were used at the next stage as a platform for constructing the representative maps. When these maps were constructed, the transcription and translation levels were determined for each construct type with specific promoter and ribosome binding site variants (Fig. 4). Further analysis allowed one to estimate the most efficient and inefficient combinations contained in the resulting construct library (Table 2) [67]. A comprehensive analysis of the variance (ANOVA) [68] of RNA and protein levels determined independently by both the promoter and the ribosome binding site was carried out. This approach also helped one to make allowance for the effects showing the association between the RNA level and the translation rate.

The programs written in R [69] and Python [70] and adapted to working with large datasets were used to visualize the resulting estimates. The ANOVA data made it possible to attribute the differences in RNA levels to the choice of promoter in 92.5% of cases, the choice of ribosome binding site in 3.8% of cases, while the remaining 3.7% of the differences could not be attributed to the choice of a variable element. The differences in the GFP protein levels were attributed to the promoter choice in 53.8% of the cases; the RBS choice, in 29.6% of cases; and the remaining percentage could be attributed to none of these two variable factors. Therefore, it was inferred that promoter choice had the greatest effect on the RNA level, while having a smaller impact on the protein level, since the translation efficiency is also affected by the choice of the ribosome binding site and, presumably, other factors as well [67].

**Table 2:** Examples of the sequences of promoters and the ribosome binding sites (RBS) ensuring inefficient and efficient expression

No.	Expression efficiency	Promoter	RBS
1	Inefficient expression	GGCGCGCCTCGACATTTATCCCTTGCGGCGA ATACTTACAGCCATAGCAA	CACCATACACATATG
2		GGCGCGCCCTGATAGCTAGCTCAGTCCTAGG GATTATGCTAGCAGATG	ATCTTAATCTAGCGCGGGACAGTTTCATATG
3		GGCGCGCCTCGACAATTAATCATCCGGCTCG ATACTTACAGCCATCGATT	TCTAGAGAAAGACCCGAGACACCATATG
4		GGCGCGCCCACGGTGTTAGACATTTATCCCTT GCGGCGAATACTTACAGCCATGTGAA	ATCTTAATCTAGCTTTGGAGTCTTTCATATG
5		GGCGCGCCTTGACAGCTAGCTCAGTCCTAGG GATTGTGCTAGCCAATC	TCTAGAGAAAGATTAGAGTCACCATATG
6		GGCGCGCCCACGGTGTTAGACAATTAATCAT CCGGCTCGATACTTACAGCCATGATTC	ATCTTAATCTAGCCCGGGAGCATTTCATATG
7		GGCGCGCCTCGACATCAGGAAAATTTTTCTG ATACTTACAGCCATGCGGA	TCTAGAGAAAGACAGGACCCACCATATG
8		GGCGCGCCCACGGTGTTAGACATCAGGAAAA TTTTTCTGATACTTACAGCCATCGACC	TCTAGAGAAAGAGCCGACATACCATATG
9		GGCGCGCCTTTATAGCTAGCTCAGCCCTTGGT ACAATGCTAGCGCCTG	ATCTTAATCTAGCCTGGGATCGTTTCATATG
10		GGCGCGCCTTTATGGCTAGCTCAGTCCTAGGT ACAATGCTAGCCATAC	ATCTTAATCTAGCCCAGGAACGTTTCATATG
1	Efficient expression	GGCGCGCCTTGACATCGCATCTTTTTGTACCT ATAATGTGTGGATAGAGT	AATCTCATATATCAAATATAGGGTGGATCA TATG
2		GGCGCGCCAAAAAGAGTATTGACTTCAGGAA AATTTTTCTGTATAATGTGTGGATGTTCA	AATCTCATATATCAAATATAAGGCGGATCA TATG
3		GGCGCGCCAAAAAGAGTATTGACTATTAATC ATCCGGCTCGTATAATAGATTCATTGAAG	ATTAAAGAGGAGAAATTACATATG
4		GGCGCGCCTTGACATCGCATCTTTTTGTACCT ATAATAGATTCATGATGA	AAAGATCTTTTAAGAAGGAGATATACATATG
5		GGCGCGCCTTGACATAAAGTCTAACCTATAG GATACTTACAGCCATACAAG	AAAGAGGAGAAATTACATATG
6		GGCGCGCCTTGACATCAGGAAAATTTTTCTG TAGATTTAACGTATAGGTA	AATCTCATAAATCAAATATAAGGGGGATC ATATG
7		GGCGCGCCAAAAAGAGTATTGACTTCGCATC TTTTTGTACCTATAATAGATTCATTGCTA	GAATTCATTAAAGAGGAGAAAGGTCATATG
8		GGCGCGCCAAAAAGAGTATTGACTTCGCATC TTTTTGTACCCATAATTATTTCATTCACA	AATCTCATATCTCAAATATAAGGGGGATCA TATG
9		GGCGCGCCAAAAAATTTATTTGCTTTTTATCC CTTGCGGCGATATAATAGATTCATCTTAG	AATCTCATAGATCAAATATAGGGGGGATC ATATG
10		GGCGCGCCAAAAAATTTATTTGCTTTCGCAT CTTTTTGTACCTATAATGTGTGGATAATAA	ATCTTAATCTAGCGGGGGAGAATTTCATATG

Note: examples of combinations of the promoter and ribosome binding site sequences were selected with allowance for the maximum and minimum RNA and translation levels, respectively, for efficient and inefficient protein expression; the sequences of restriction sites are underlined; the last five nucleotides in the promoter sequences act as the unique barcode for identification of the transcription initiation site. The sequences are shown in the 5’→3’ orientation.

A number of studies employing the Flow-seq method have investigated the effect of the sequences of 5’ untranslated regions of different lengths and their individual sites on the efficiency of the reporter fluorescent protein synthesis [2, 39, 71, 72, 73, 74].

Variation in the spacer regions residing between the Shine–Dalgarno sequence and the start codon enabled the construction of small-sized libraries, where four and six nucleotides in a given site were randomly generated. A 100- [39] and 1,000-fold [71] difference between the highest and lowest produced protein level, respectively, was successfully obtained. In the former case, the most efficient and inefficient sequences included the following spacer sequences: cAAAAcau, cGAAAcau, cAUAAcau, cAUAUcau and cCCGCcau, cCUCUcau, cCGCUcau, cCCGUcau, respectively, by SD sequence (GAGG) flanking at the 5’ terminus and by the start codon (AUG) at the 3’ terminus. In the latter case, among the sequences residing downstream of the SD sequence (AAGAAGGA) and upstream of the start codon (AUG) and ensuring the highest expression, one can distinguish the gacUAGAGC, gacUGUAAG, gacAAAACC, and gacGUGGUU sequences. Interestingly, the CAAAAC sequence emerges as one of the most effective sequences in both cases.

In the former case, single-stranded oligonucleotides with four random nucleotides in the spacer region and the restriction sites required for subsequent insertion of the fluorescent protein CER gene into the vector upstream of the start codon were used for library generation. The resulting set of cells was subjected to sorting, and the selected variable plasmid regions were used for next-generation sequencing [39].

In the latter case, to optimize the synthesis of two specific proteins encoded by the araHWT and narKWT genes, their coding sequences were bound to the region encoding the TEV-GFP-His8 additional sequence, where TEV is the recognition site of the tobacco etch virus protease (BTM/TEV); His8 is a tag composed of eight His residues for further purification. Therefore, the measured GFP fluorescence can be indicative of the expression levels of the genes of interest. A vector comprising the aforedescribed complex coding region under the control of the T7 promoter, and two primers (the reverse one being invariant and the forward one containing six variable nucleotides upstream and downstream of the start codon; these nucleotides met the criteria of synonymous codon substitutions) was used for library construction. Expression was induced by IPTG; the cells were then sorted into separate fractions by FACS according to the intensity of the GFP protein fluorescence. Plasmid DNA libraries were then isolated from these fractions and subjected to high-throughput sequencing [71].

An analysis of the sequencing data for several tens of thousands of different mRNA variants obtained in the two experiments described above showed that the low GC-content and the absence (or minimization) of the mRNA secondary structure in the spacer region under study increased the amount of the synthesized protein [39, 71]. Therefore, it seems reasonable to use oligoadenylate or other A-rich spacers between the SD sequence and the start codon to increase the protein synthesis yield, while avoiding the use of cytidine bases, although one should not rule out certain specific mRNAs with A-rich spacer regions, which can mask the translation initiation site in their secondary structure if the beginning of the coding region is U-rich.

These results should be taken into account when designing reporter plasmids when there is a need for the expression levels of exogenous genes to be tuned according to specific biotechnological needs. For the coexpression of the genes whose products are supposed to be synthesized in a given stoichiometric ratio (e.g., when proteins are subunits of the heteromultimeric complex), the expression levels of these genes can be regulated by a judicious choice of the spacer regions.

Determining the sensitivity to minor variations in the sequence of the regulatory elements in the 5’ UTR, such as the Shine–Dalgarno sequence, is rather challenging, since minor variations in the 5’ UTR may lead to unpredictable changes in the gene expression level [34, 75]. The dependence of the translation efficiency on the 5’ UTR sequence enables efficient and multiplex engineering, provided that the models being built can adequately predict these changes [73].

EMOPEC (Empirical Model and Oligos for Protein Expression Changes), another tool for predicting gene expression levels in bioengineering, has been developed; it is a nearly complete database of gfp expression levels measured using the Flow-seq method, depending on the presence of a particular SD sequence [76].

It is well known that the effect of a particular SD sequence largely depends on its genetic context [32]. Accordingly, special care should be taken when reapplying the measured expression levels in the bioengineering of metabolic pathways or synthetic biology, since the ribosome binding site depends in large part on the local secondary structure of mRNA. However, whereas the Shine–Dalgarno sequences can be modified by making minimal changes to the secondary structure in a given mRNA region, the relative order of expression level of a particular SD sequence will probably remain intact [73]. These features are taken into account when using the algorithm in the EMOPEC database, which allows one to test a wide range of gene expression levels, with minimal changes in the SD sequence. Therefore, parallel and efficient genome editing tuning gene expression levels becomes possible.

The Flow-seq method has been repeatedly used to gain insight into how the nucleotide sequences of different motifs of 5’ UTRs affect the translation efficiency. In particular, the ribosome binding sites with a fixed SD sequence [74], 5’ UTRs of different fixed lengths [2], or natural 5’ UTRs of different lengths [77], as well as standby sites and spacer regions [72], were studied. An analysis of tens of thousands of tested variants showed that the variation in the efficiency of the reporter protein synthesis can amount to four, and even five, orders of magnitude. Moreover, replacement of one reporter protein with another one often did not affect the general trend of sequence distribution, which sets a particular level of protein biosynthesis, indicating that these changes are determined specifically by variable mRNA regions. Similar observations relating to the low stability of the secondary structure and the conservation of the SD sequence were made for the variants determining a high translation efficiency [2]. The same factors were found to be significant for the translation efficiency of the reporter gene preceded by a set of natural 5’ UTRs; however, in this case, the variability of the translation efficiency was much lower than it was for the library of fully randomized 5’ UTR sequences [77].  There were also individual cases being indicative of the presence of AU-rich enhancers at the 5’ terminus at the standby site, low abundance of cytidine bases, multiple SD sequences, and AG repeats in the mRNA 5’ UTRs, which provide the high reporter protein level [2].

A similar approach was also used to elucidate the effect of rare codons at the beginning of the mRNA coding region on the translation efficiency [78]. According to observations, rare codons are more frequently found at the beginning of the coding region of natural genes, especially the highly expressed ones, which may be important for ensuring the high protein synthesis level [64, 79 , 80, 81, 82]. According to other data, codon rarity at the beginning of the coding region is simply a consequence of a selection driven by the urge to minimize the secondary structure at the beginning of the mRNA coding region [19, 78, 82]. In the research literature, there is an ongoing discussion about the causes and consequences of rare codon clusters at the beginning of coding regions and how these clusters affect the translation efficiency. The potential reasons for the diverging opinions can lie in the collection peculiarities of the data on which these opinions are based. In particular, different research groups used natural [79, 80, 81, 82, 83, 84] or synthetic sequences [80, 85, 86, 87, 88, 89, 90], as well as slightly different methods of analysis [79, 80, 81, 82, 83, 84, 85, 86, 87, 88, 89, 90], in drawing their conclusions.

In order to elucidate the reasons for the increased abundance of rare codons at the beginning of the coding region of bacterial genes and its functional role, a large library comprising 14,234 combinations of two promoters (strong and weak ones), four ribosome binding sites (strong, medium, weak, and natural ones), and sequences of the first 13 codons of 137 E. coli genes was constructed based on an oligonucleotide array. These regulatory elements were placed upstream of the gene encoding the super-folder green fluorescent protein (sfGFP) in the plasmid from which the mCherry protein is constitutively coexpressed [78]. The DNA, RNA, and protein levels were measured in the entire constructed library using DNA-seq, RNA-seq, and Flow-seq, respectively

According to the “codon ramp” hypothesis, the first N-terminal codons in the coding region are slowly translated, which subsequently reduces ribosome stalling during protein synthesis [79, 88, 89]. The increase in the translation efficiency in the presence of rare codons at the beginning of the coding region can be attributed to changes in the mRNA secondary structure rather than to codon rarity [78]. Finally, the ribosome occupancy profiles have demonstrated that tRNA concentration, which actually is responsible for the efficiency of codon usage, does not correlate with the translation rate. Specific rare codons can create internal motifs similar to the SD sequence; in turn, they can affect the translation efficiency in E. coli cells [64]. Searching for an association between the internal SD-like motifs and variations in expression has revealed a weak but statistically significant relationship.

A study focusing on the effect of synonymous mutations on the translation efficiency has led to the following conclusion: the presence of rare codons in E. coli, often A/T-rich at position 3, is more likely to correlate with increased expression than the presence of synonymous G/C-ending codons, being indicative of an association with the mRNA secondary structure [85]. It has also been shown that reduction of GC content correlates with increased protein expression [78]. By predicting the RNA secondary structure for the first 120 bases of each transcript using the NUPACK software specializing in nucleic acid folding [91], it was found that the increase in strength of the secondary structure correlated with a reduction in the expression level, which explained why variation was more significant than any other change assessed previously [78].

More than 30 × 10^3^ codon variants at positions 2–11 of the coding region of the reporter fluorescent protein obtained by randomization of the first 30 nucleotides downstream of the start codon were subsequently analyzed. The gene encoding the second fluorescent protein remained unchanged and was used as an internal control. The constructed plasmid library was examined using the Flow-seq method [28], making it possible to confirm that the mRNA secondary structure has a negative effect on the translation efficiency, while no positive role of the rare codons at the beginning of the coding region in gene expression was observed.

Meanwhile, the following patterns have been revealed. Some codons residing at the beginning of the coding region have a positive (AUG, AGA, GUA, GCA, CAC, CGA, UAC, AAA encoding additional Met along with the initiator one, the positively charged amino acids Arg, Lys, His, hydrophobic aliphatic Ala, Val and aromatic Tyr), while some others have a negative (CUC, CCC, CCG, CUG, GGA, GGG, GGC, GCC encoding hydrophobic aliphatic amino acids and amino acids with more or less conformational freedom compared to the rest of the amino acids Leu, Pro, Gly, and Ala) effect on the expression level. The closer the respective codon is to the initiator codon, the stronger the influence it has. Additional start codons in the reading frame facilitate translation. The presence of amino acids (the cell spends a lot of resources for synthesizing them) in the N-terminal motif of the protein negatively affects the synthesis efficiency of such proteins in a depleted environment.

Application of the Flow-seq method is not limited to the provided examples. This technique is also employed to evaluate (using reporter constructs as biosensors in various bacterial strains, including knockout ones [92]) the effects on the glycolytic processes, assess terminator sequences [93], identify the genes involved in the changes in a particular metabolic pathway (using biosensor constructs [94]), and solve other problems (e.g., study splicing) [95].

## THE CONTRIBUTION OF THE FLOW-seq METHOD TO SYNTHETIC BIOLOGY

Synthetic biology is a recent scientific discipline that deals with designing and creating living organisms or individual processes occurring in natural organisms [96, 97, 98]. This discipline has emerged and has been developing through a combination of genetic engineering and recombinant DNA technologies with computational modeling. Therefore, synthetic biology seeks to identify the behavior of organisms and the processes occurring in them in order to subsequently modify and combine them to solve complex specific problems. For synthetic multicomponent systems to work reliably, the proteins comprising the system need to form at customized ratios [97].

Three calculator programs have been developed for estimating the translation efficiency based on the 5’ UTR mRNA sequences, since the overall translation rate is believed to be proportional to the translation initiation rate. These calculators were shown to adequately estimate the protein synthesis level.

The RBS Calculator was the first one to appear among the three calculators [33, 99]. It relied upon the thermodynamic model studied previously and was a predictive design method for ensuring controlled translation initiation and protein synthesis in bacteria [32, 33]. This method allows one to vary the translation efficiency within the range of five orders of magnitude [33, 34]. However, the predictions made using the RBS Calculator are not always consistent with the experimental data obtained by Flow-seq or by testing individual reporter constructs [2].

The UTR Designer (or UTR Library Designer) is another computational method for modeling 5’ UTR sequences capable of predicting the translation efficiency according to the mRNA sequence carrying a particular 5’ UTR [100, 101]. Being similar to the RBS Calculator, this method employs a thermodynamic parameter defined as the difference in the Gibbs free energies before and after the assembly of the 30S translation initiation complex on mRNA and takes into account the affinity of ribosome interaction, as well as the availability of mRNA and ribosome. Like the RBS Calculator, this software has two engineering modes: in the forward-engineering mode, it generates a 5’ UTR with a specified translation efficiency level of the target protein sequence. In the reverse-engineering mode, the calculator predicts the level of protein synthesis from the inserted mRNA sequence carrying the 5’ UTR and the first 35 nucleotides of the protein-coding region. The operational principle of the described method of constructing the mRNA library with different 5’ UTRs is to generate 5’ UTR sequences by generating random nucleotide sequences and combinatorial enumeration of construction variants with a choice of those capable of providing the desired protein translation level. Moreover, there is a constant portion of the 5’ UTR which must be present in the resulting sequence: in this case, the combinatorial enumeration will refer exclusively to its environment. This method was validated for two libraries of 5’ UTRs carrying 16 sequences characterized by different translation levels lying in a given range using a fluorescent reporter; the in silico predictions agreed well with the in vivo data [100]. However, the predictions made using this approach are sometimes far from correlating with the in vivo results obtained for other 5’ UTR sequence samples in the selected range of protein synthesis efficiencies.

Like the previous two calculators, the third one, RBS Designer, calculates the free energies but differs in the method used for predicting the translation rate. Relying on the steady-state kinetic model, this calculator estimates the probability of binding between a particular mRNA and the ribosome (translation efficiency), according to the chances for availability of the RBS-carrying mRNA region and affinity of ribosome binding. Each calculator is characterized by similar prediction accuracy [97].

Several prediction models have been reported thus far. They were constructed due to the vast amount of data obtained by large-scale sequencing, the analysis of various libraries, and the findings obtained using other genetic engineering techniques. A good example is the potential prediction of translation initiation sites, which is useful for localizing protein-coding gene sites during computer-assisted annotation of bacterial and archaeal genomes [102], and prediction of putative genomic sequences that correspond to functional RNA motifs [103], or prediction of gene expression levels with new combinations of genetic elements [75].

Even experimental verification of the translation efficiency determined by any binding site in a model system cannot guarantee that an identical efficiency will be achieved if the coding region sequence is replaced. Such is the case due to secondary structure formation when the coding region and the 5’ UTR are complementary. A study using specially designed bicistronic constructs was conducted in order to increase the predictability of the expression level of any gene expressed in a heterologous system. In that study, a conventional short open reading frame was located upstream of the reporter coding region whose expression efficiency was measured by flow cytometry. The reading frames overlapped within the randomized translation re-initiation site. Therefore, it was found that re-initiation eliminates the dependence of the translation efficiency on the coding region of the second gene. Both gfp and rfp were used as the second gene in this synthetic operon. The resulting expression levels of these different genes correlated well with each other [75].

Hence, experimental determination of the expression efficiency by flow cytometry or Flow-seq can be directly and reliably employed for generating expression constructs in synthetic biology.

## CONCLUSIONS

The Flow-seq technique combines flexible genetic bioengineering approaches and cell sorting based on flow cytometry and high-throughput sequencing of DNA to comprehensively assess genotype–phenotype associations. One of the applications of Flow-seq is in the study of the effect of specific regulatory elements on protein synthesis (Table 1). Designing tailored changes based on reporter constructs using the fluorescent protein genes allows one to quickly and efficiently elucidate the contribution of specific variants of regulatory sequences to the protein synthesis efficiency. Like other methods used to study the effect of 5’ untranslated region elements in mRNA on the translation efficiency, this approach has its own peculiarities that should be taken into account when planning a complex multi-step experiment. Although the method discussed in this review has great potential, its application has some limitations, primarily caused by the challenges arising at different stages, such as DNA library cloning, sorting of cells with different ratios of fluorescence intensities of the reporter proteins, high-throughput sequencing, analysis of the reads obtained, and further calculations. Another limitation is that only two fluorescent proteins or other detectable reagents of such type are used, since there is a risk of fluorescence spectral overlapping for these proteins and, therefore, signal registration errors. Nonetheless, the Flow-seq method is widely used in various research fields and has remained relevant for many years.

## Abbreviations

TIR: translation initiation region
RBS: ribosome binding site
SD: Shine-Dalgarno sequence
5’ UTR: 5’ untranslated region
ORF: open reading frame
NGS: next-generation sequencing
Flow-seq: flow cytometry and next-generation sequencing
